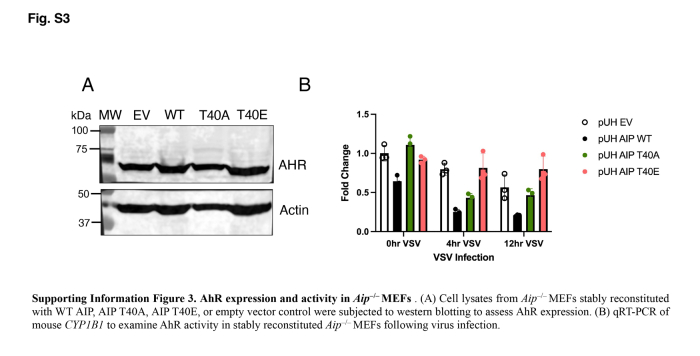# Phosphorylation of aryl hydrocarbon receptor interacting protein by TBK1 negatively regulates IRF7 and the type I interferon response

**DOI:** 10.1016/j.jbc.2023.105525

**Published:** 2023-12-01

**Authors:** Sarah A. Kazzaz, Kashif A. Shaikh, Jesse White, Qinjie Zhou, Wade H. Powell, Edward W. Harhaj

**Affiliations:** 1 Department of Microbiology and Immunology, Penn State College of Medicine, Hershey, Pennsylvania, USA; 2 Medical Scientist Training Program, Penn State College of Medicine, Hershey, Pennsylvania, USA; 3 Department of Microbiology and Immunology, Miller School of Medicine, The University of Miami, Miami, Florida, USA; 4 Biology Department, Kenyon College, Gambier, Ohio, USA

**Keywords:** aryl hydrocarbon receptor interacting protein (AIP), interferon regulatory factor 7 (IRF7), TANK binding kinase 1 (TBK1), type I interferon, antiviral signaling

## Abstract

The innate antiviral response to RNA viruses is initiated by sensing of viral RNAs by RIG-I-like receptors and elicits type I interferon (IFN) production, which stimulates the expression of IFN-stimulated genes that orchestrate the antiviral response to prevent systemic infection. Negative regulation of type I IFN and its master regulator, transcription factor IRF7, is essential to maintain immune homeostasis. We previously demonstrated that AIP (aryl hydrocarbon receptor interacting protein) functions as a negative regulator of the innate antiviral immune response by binding to and sequestering IRF7 in the cytoplasm, thereby preventing IRF7 transcriptional activation and type I IFN production. However, it remains unknown how AIP inhibition of IRF7 is regulated. We show here that the kinase TBK1 phosphorylates AIP and Thr40 serves as the primary target for TBK1 phosphorylation. AIP Thr40 plays critical roles in regulating AIP stability and mediating its interaction with IRF7. The AIP phosphomimetic T40E exhibited increased proteasomal degradation and enhanced interaction with IRF7 compared with wildtype AIP. AIP T40E also blocked IRF7 nuclear translocation, which resulted in reduced type I IFN production and increased viral replication. In sharp contrast, AIP phosphonull mutant T40A had impaired IRF7 binding, and stable expression of AIP T40A in AIP-deficient mouse embryonic fibroblasts elicited a heightened type I IFN response and diminished RNA virus replication. Taken together, these results demonstrate that TBK1-mediated phosphorylation of AIP at Thr40 functions as a molecular switch that enables AIP to interact with and inhibit IRF7, thus preventing overactivation of type I IFN genes by IRF7.

The innate immune system is a key component of host immunity and serves as the first line of defense responsible for recognizing and mounting responses to invading pathogens. Viral pathogen–associated molecular patterns activate host pattern recognition receptors, triggering the induction of type I interferon (IFN) production (1). Type I IFNs (IFN-α/β) activate the transcription of interferon-stimulated genes, which are crucial for the restriction of viral replication and spread, and stimulate the adaptive immune system (2, 3).

RNA viruses are detected by either the cytoplasmic retinoic inducible gene 1 (RIG-I)-like receptors (RLRs) or the endosomal Toll-like receptors. The DExD/H box RNA helicases RIG-I and melanoma differentiation-associated protein 5 (MDA5) recognize 5′-triphosphate single-stranded RNA and double-stranded RNA, respectively (4, 5). The amino terminus of RIG-I contains a Caspase activation and recruitment domain (CARD), and the carboxyl terminus contains an RNA helicase domain (6). Upon viral infection, the CARD domain undergoes tripartite motif containing 25–mediated Lys63 (K63)-linked polyubiquitination, which allows it to interact with the CARD domain of mitochondrial antiviral signaling protein (MAVS), which is anchored to the outer mitochondrial membrane (7). MAVS then forms prion-like aggregates and recruits a signaling complex of tumor necrosis factor receptor–associated factor (TRAF) E3 ligases (TRAF2, TRAF3, TRAF5, and TRAF6), NF-kappa-B essential modulator, and the serine/threonine kinases TBK1 and IKKε (inhibitor of nuclear factor kappa B kinase, subunit epsilon) (also known as IKKi) (8). TBK1 and IKKi phosphorylate IRF3 and IRF7 transcription factors, leading to their dimerization and translocation to the nucleus where they induce type I IFN expression (9, 10).

The IRF family of proteins are transcriptional regulators of the interferon-stimulated response element. IRF3 and IRF7 are the principal mediators of IFN induction, and while they share significant structural homology, their roles in generating an immune response differ (11). IRF3 is ubiquitously expressed and is primarily responsible for the initial induction of type I IFNs. In contrast, basal expression of IRF7 is low (except in plasmacytoid dendritic and lymphoid cells) and, as an interferon-stimulated gene, IRF7 expression is induced following the initial wave of type I IFN production (11). In addition, IRF7 is responsible for a larger-scale production of type I IFN and consequently *Irf7*^−/−^ mice are highly susceptible to RNA virus infections (12). For these collective reasons, IRF7 is considered the master regulator of type I IFN.

While activation of the innate immune system is essential for the host response to viral infections (13, 14), downregulation of RLR and type I IFN signaling is important to maintain immune homeostasis and prevent excessive or chronic inflammation. As such, aberrant type I IFN activation and/or signaling has been linked to viral cytokine storms (*i.e.*, influenza A virus and COVID-19) (15, 16) and autoimmune diseases. Overactivation of IRF7 has been implicated in systemic lupus erythematosus, scleroderma, and type I diabetes (17, 18, 19). Although the mechanisms of negative regulation of RLR signaling have been well described (1, 20, 21, 22, 23), how IRF7 is negatively regulated remains poorly understood.

AIP (aryl hydrocarbon receptor interacting protein, also known as XAP2, ARA9, and FKBP37) encodes a 330-amino-acid protein and contains an N-terminal peptidyl-prolyl *cis-trans*-isomerase (PPIase)-like domain, three tetratricopeptide repeat (TPR) domains, and an alpha-7-helix at its C terminus. The PPIase domain shares homology with the FKBP family of chaperone proteins that function as “molecular switches” involved in protein folding and cellular signaling (24, 25, 26). AIP was first identified as a co-chaperone for AhR (along with heat shock protein 90) and was thought to stabilize AhR in its inactive conformation within the cytoplasm (27). Upon ligand binding, AhR sheds its co-chaperone proteins and translocates into the nucleus, binding to the xenobiotic response element to upregulate several genes, including the cytochrome P450 family of enzymes. While AhR was originally thought to regulate metabolic responses, its roles have expanded to cell growth, cell migration, apoptosis, hematopoiesis, carcinogenesis, and immune regulation (27, 28).

Similar to those of AhR, the functional roles of AIP have greatly expanded beyond AhR regulation and the xenobiotic response. Emerging studies have described new and unanticipated roles of AIP in the regulation of the immune system. AIP has been shown to interact with viral proteins EBNA-3 of Epstein–Barr virus (29) and the X protein of hepatitis B virus (30). AIP was also found to bind to and positively regulate the CARMA1–BCL10–MALT1 complex for T-cell activation (31). In germinal centers, AIP prevents B-cell lymphoma protein 6 proteasomal degradation through its interaction with the deubiquitinating enzyme, ubiquitin carboxy-terminal hydrolase L1, which promotes B-cell proliferation (32). Furthermore, our previous study demonstrated that AIP binds to IRF7 and prevents its nuclear translocation, thus acting as a negative regulator of innate antiviral signaling (33). However, the mechanisms underlying AIP regulation of IRF7 remain unknown. In this study we demonstrate that TBK1 phosphorylates AIP on residue Thr40 (T40) and this phosphorylation event plays an essential role in the inhibition of IRF7 and type I IFN.

## Results

### TBK1 phosphorylates AIP at T40, S131, and S132

**Figure 1:**
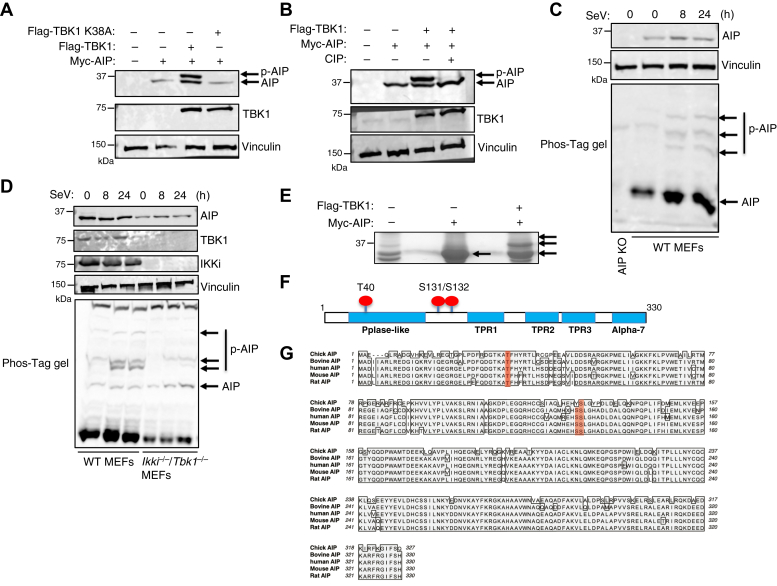
**TBK1 phosphorylates AIP.** Western blot analysis of: (*A*) 293T cells transfected with Myc-AIP alone or cotransfected with Flag-TBK1 or kinase dead Flag-TBK1 K38A; (*B*) 293T cells transfected with Myc-AIP alone or cotransfected with Flag-TBK1, followed by treatment with vehicle control or calf intestinal phosphatase (CIP) (1 unit/μg of protein) for 1 h. *C* and *D*, WT and *Ikki*^−/−^*Tbk1*^−/−^ MEFs were infected with SeV for 0, 8, or 24 h. Lysates were subjected to Western blotting (*top*) and Phos-Tag gels (*bottom*) to identify phosphorylated proteins; (*E*) 293T cells were transfected with Myc-AIP and/or Flag-TBK1, and lysates were subjected to mass spectrometric analysis. Arrows indicate the bands excised for mass spectrometry. *F*, schematic of the three putative TBK1 phosphorylation sites: T40, S131, and S132 identified by LC-MS/MS analysis. *G*, sequence alignment of human, mouse, rat, chicken, and bovine AIP. The three putative TBK1 phosphorylation sites are highlighted in *red*.

Our previous study demonstrated that AIP inhibits IRF7 (33); however, how AIP inhibition of IRF7 is regulated remains unknown. During the course of our studies, we noticed that overexpression of TBK1 impaired the mobility of AIP on SDS-PAGE gels, causing a band shift indicative of a phosphorylated form of AIP (Fig. 1*A*). However, the band shift of AIP was not evident when AIP was cotransfected with kinase dead TBK1 K38A (Fig. 1*A*). We confirmed that the band shift was due to phosphorylation since treatment of protein lysates with calf intestinal phosphatase reversed the slower migrating band of AIP (Fig. 1*B*).

**Supplemental Figure S1:**
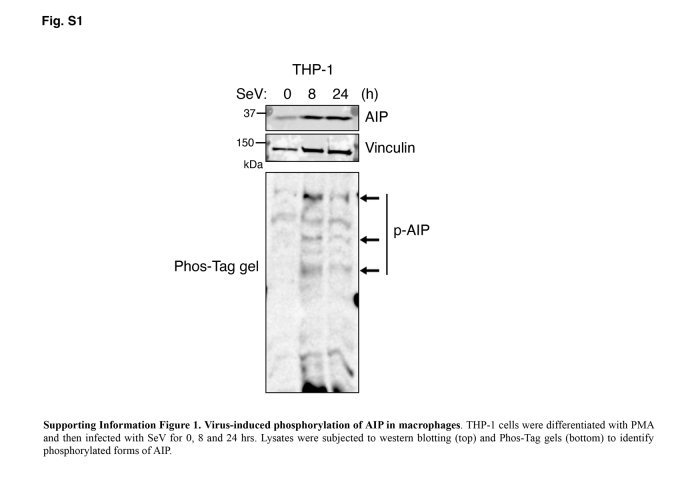


Since TBK1 is activated by RLR signaling during RNA virus infection (34), we hypothesized that TBK1 phosphorylates AIP during virus infection as part of a negative feedback mechanism. To begin testing this hypothesis, mouse embryonic fibroblasts (MEFs) were infected with an RNA virus, Sendai virus (SeV), and phosphorylation of endogenous AIP was examined using Phos-Tag gels. Phosphorylated proteins undergo large mobility shifts in Phos-Tag gels, which can also provide insight about the stoichiometry of phosphorylation events (*i.e.*, multisite phosphorylation) (35). Three distinct shifted bands of AIP were induced 8 and 24 h after SeV infection (Fig. 1*C*), suggesting multisite phosphorylation of AIP. Virus-mediated AIP phosphorylation was not specific to MEFs and was also observed in the human monocytic cell line THP-1 (Fig. S1). To determine if TBK1 was required for virus-induced phosphorylation of endogenous AIP, *Ikki*^−/−^*Tbk1*^−/−^ MEFs were infected with SeV since IKKi could potentially compensate for the loss of TBK1 (36). *Ikki*^−/−^*Tbk1*^−/−^ MEFs did not exhibit any phosphorylated AIP band shifts after virus infection (Fig. 1*D*), indicating a requirement for TBK1/IKKi in phosphorylating AIP following RNA virus infection.

To identify TBK1-induced AIP phosphorylation sites in an unbiased manner, we next performed liquid chromatography/tandem mass spectrometry (LC-MS/MS) using whole cell lysates from cells transfected with AIP and TBK1 (Fig. 1*E*). This approach yielded three putative TBK1-induced phosphorylation sites in AIP: T40, Ser131 (S131), and Ser132 (S132) (Fig. 1*F*). T40 is within the PPIase domain, whereas S131 and S132 are located within the linker between the PPIase and TPR1 domains. All three of these residues are highly conserved across several species with only slight variation in S131, which is a tyrosine in chickens (Fig. 1*G*). These results suggest that TBK1 may phosphorylate AIP at T40, S131, and S132.

### AIP T40 is the primary site of TBK1 phosphorylation and regulates protein stability

**Figure 2:**
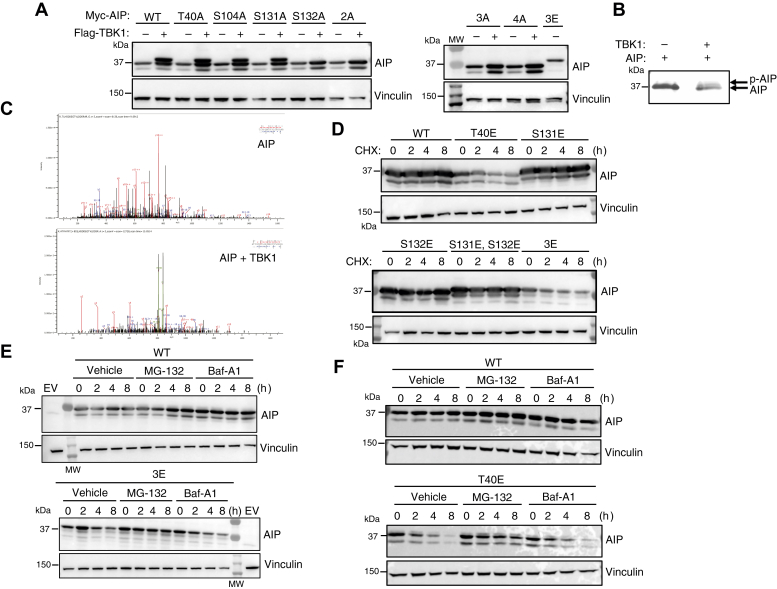
**T40 is the major TBK1-induced AIP phosphorylation site and regulates AIP stability.***A*, Western blot analysis of 293T cells transfected with WT Myc-AIP or Myc-AIP phosphonull mutants alone or cotransfected with Flag-TBK1. 2A indicates AIP S131A/S132A; 3A is AIP T40A/S131A/S132A; 4A is AIP T40A/S104A/S131A/S132A. AIP 3E (T40E/S131E/S132E) serves as a positive control for the band shift in AIP that suggests that AIP is phosphorylated. *B*, immunoblot analysis of an *in vitro* kinase assay of recombinant AIP alone or with recombinant TBK1. *C*, mass spectrometry results of recombinant AIP alone (*top*) *versus* recombinant AIP plus recombinant TBK1 (*bottom*) indicate there is an inducible phosphorylation modification of AIP T40 by TBK1. *D*, cycloheximide chase assay of 293T cells transiently transfected with WT Myc-AIP or phosphomimetic mutants for 24 h, then treated with cycloheximide (100 μg/ml) for the indicated times. *E* and *F*, cycloheximide chase assay of 293T cells transiently transfected with WT Myc-AIP, Myc-AIP 3E, or Myc-AIP T40E with vehicle control (dimethyl sulfoxide), proteasome inhibitor (MG-132, 10 μM), or lysosome inhibitor (Baf-A1, 100 nM).

We next examined the potential contributions of AIP T40, S131, and S132 to the band shift caused by TBK1 overexpression. Phosphonull mutants were generated by mutating the residues to alanine, creating constitutively inactive forms of AIP. Constitutively active, phosphomimetic forms of AIP were generated by mutating the residues to glutamic acid (E). Single, S131/S132 double (termed 2A and 2E), and T40/S131/S132 triple (termed 3A and 3E) mutants were generated. Since S104 represented another potential TBK1 phosphorylation site identified by bioinformatics analysis (NetPhos 2.0; data not shown), S104A and a quadruple mutant including S104 (T40A/S104A/S131A/S132A termed 4A) were also generated. The panel of AIP single and compound mutants was examined for TBK1-induced phosphorylation as assessed by a band shift on SDS-PAGE gels. This experiment revealed that AIP S132 was responsible for the observed TBK1-induced band shift since S132A was impaired for the band shift (Fig. 2*A*). Together, these results indicate that TBK1 may phosphorylate multiple sites in AIP but only S132 contributes to the band shift of AIP.

To determine if TBK1 directly phosphorylates AIP and to identify direct phosphorylation sites, we performed an *in vitro* kinase assay with recombinant TBK1 and AIP; this yielded a band shift of AIP on an SDS-PAGE gel suggesting that TBK1 directly phosphorylates AIP (Fig. 2*B*). The bands were then excised from the gel for LC-MS/MS analysis and identification of phosphorylation sites. No phosphopeptides were identified with AIP alone, but AIP T40 was found to be phosphorylated in the presence of TBK1 (Fig. 2*C*). No other AIP phosphorylation sites were identified suggesting that AIP T40 is likely the major and direct phosphorylation site of TBK1.

We next examined the stability of AIP phosphomimetic proteins by cycloheximide (CHX) chase assays. The AIP phosphomimetic triple mutant 3E (T40E, S131E, S132E) appeared to be less stable in CHX assays (Fig. 2*D*). Furthermore, the AIP single mutant T40E was also less stable, indicating that T40 was the key residue regulating the stability of AIP (Fig. 2*D*). To determine if the degradation of AIP 3E was mediated by the proteasome or autophagosomes/lysosomes, cells were treated with either the proteasome inhibitor MG-132 or the vacuolar H^+^-ATPase inhibitor Bafilomycin A1 (Baf-A1). AIP 3E and AIP T40E degradation was largely rescued by MG-132 treatment, indicating that AIP 3E and T40E degradation occurs through the proteasomal pathway (Fig. 2, *E* and *F*). These results suggest that T40 phosphorylation may promote AIP degradation via the proteasome.

### AIP T40 phosphorylation promotes an interaction with IRF7

**Figure 3:**
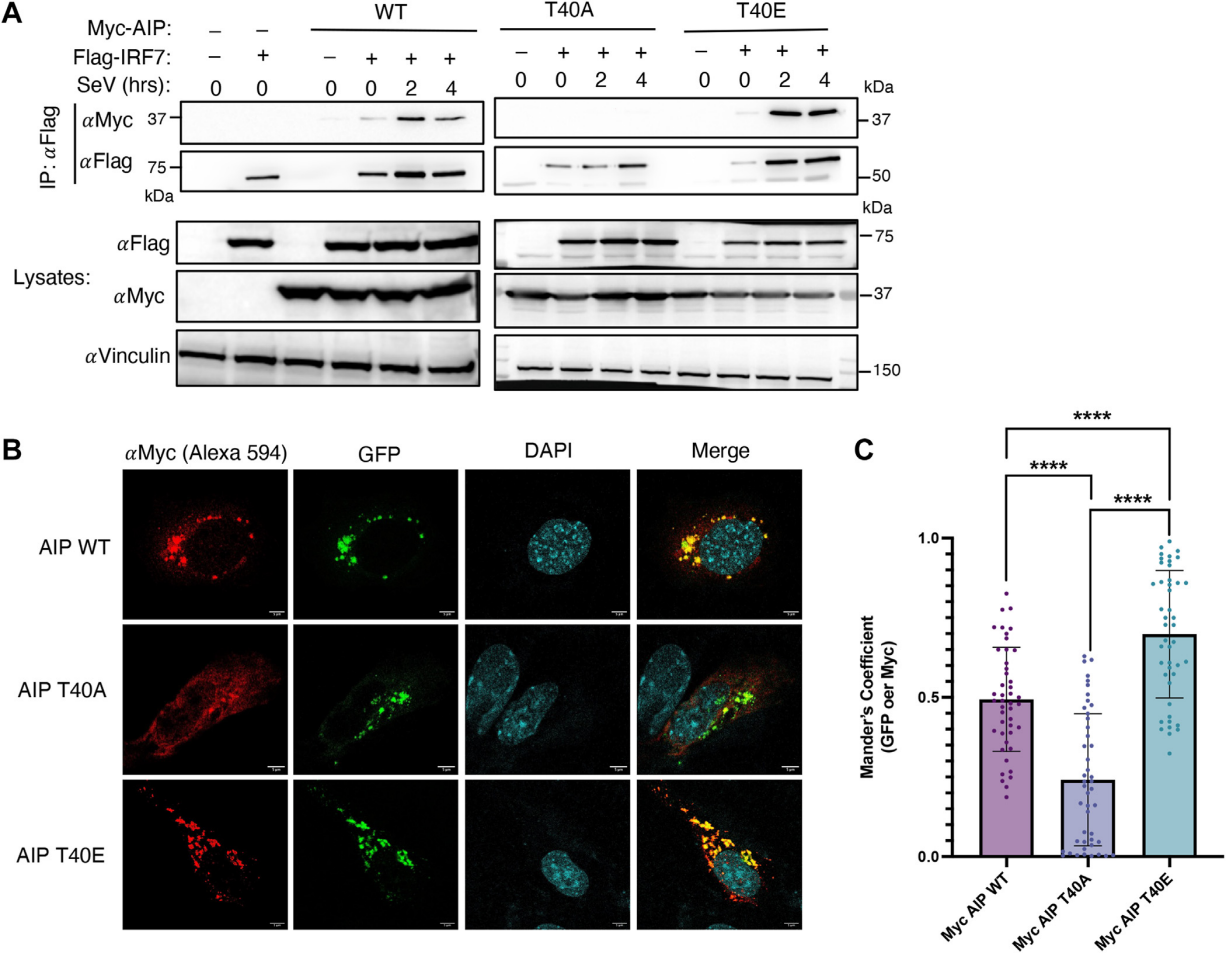
**AIP phosphorylation promotes IRF7 interaction.***A*, 293T cells were transfected with Myc-AIP and/or Flag-IRF7, and 24 h later cells were infected with SeV for the indicated times. Cells were then lysed and subjected to coimmunoprecipitation and Western blotting using the indicated antibodies. *B* and *C*, *Aip*^−/−^ MEFs were cotransfected with GFP-IRF7 and WT Myc-AIP or T40 mutant plasmids. *B*, representative confocal images of *Aip*^−/−^ MEFs cotransfected with GFP-IRF7 and Myc-AIP. *C*, quantification of Myc and GFP colocalization by Mander’s coefficient of GFP over Myc for the three different Myc-AIP plasmids. Mean Mander’s coefficient were 0.493, 0.2413, and 0.6985 for AIP WT, T40A, and T40E, respectively. *Dots* represent individual values, and error bars represent standard deviation (n = 45, ∗∗∗∗ indicates *p*-value <0.0001). Scale bars represent 5 μm.

**Supplemental Figure S2:**
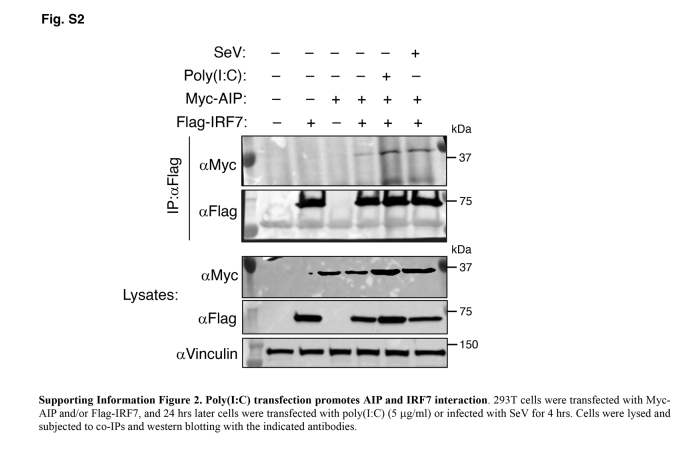


Our previous study demonstrated that AIP binds to IRF7 and this interaction is enhanced by virus infection (33). Given that AIP is inducibly phosphorylated during virus infection (Fig. 1*C*), we next assessed the interaction between IRF7 and the AIP T40 mutants by coimmunoprecipitation assays. As expected, wildtype (WT) AIP interacted with IRF7 basally, and this interaction was further increased during SeV infection (Fig. 3*A*). The phosphonull AIP T40A mutant did not interact with IRF7 either basally or during SeV infection (Fig. 3*A*). In contrast, the phosphomimetic AIP T40E mutant exhibited a more robust basal and virus-induced interaction with IRF7 compared with WT AIP (Fig. 3*A*). Transfection of the double-stranded RNA mimetic poly(I:C), which activates the RIG-I-like receptor MDA5, also enhanced the interaction between WT AIP and IRF7 (Fig. S2).

We next examined the colocalization of AIP and IRF7 by confocal microscopy. For these experiments, we transfected *Aip*^−/−^ MEFs with Myc-AIP (WT, T40A and T40E) and IRF7 fused with green fluorescent protein (GFP), GFP-IRF7. As expected, AIP was localized in the cytoplasm in a perinuclear area (Fig. 3*B*). Colocalization analysis revealed that GFP-IRF7 modestly colocalized with WT AIP (Mander’s coefficient = 0.4937) but not with AIP T40A (Mander’s coefficient = 0.2413) (Fig. 3, *B* and *C*). Consistent with coimmunoprecipitation experiments, GFP-IRF7 most strongly colocalized with AIP T40E (Mander’s coefficient = 0.6985) (Fig. 3, *B* and *C*). These results indicate that phosphorylation of T40 promotes the interaction of AIP with IRF7.

### AIP T40 phosphorylation suppresses type I IFN expression and enhances viral replication

**Figure 4:**
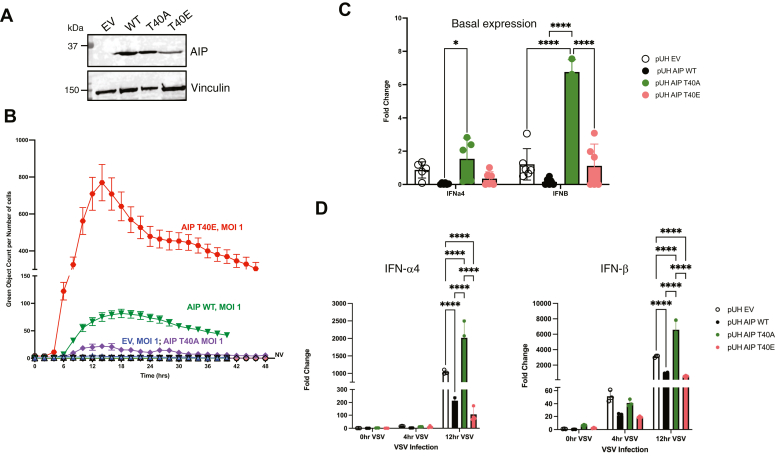
**T40 is critical for AIP to inhibit IRF7 and type I IFN.***A*, *Aip*^−/−^ MEFs were stably reconstituted with EV, WT AIP, T40A, or T40E. AIP expression was assessed by Western blotting using cell lysates. *B*, *Aip*^−/−^ MEFs stably expressing WT AIP, T40A, or T40E were infected with VSV-GFP at an MOI = 1.0. *Green* object count (representative of viral replication) was monitored by live cell imaging using an Incucyte S3. *C* and *D*, quantitative real-time PCR analysis of *Aip*^−/−^ MEFs stably expressing WT AIP, T40A, or T40E and infected with VSV-WT at MOI = 1.0 for 0, 4, or 12 h. *C*, basal levels of indicated genes, normalized to EV. *D*, fold induction of IFNα4 and IFNβ are shown and were normalized to EV 0 h VSV infection (n = 3, ∗∗∗∗ indicates *p*-value <0.0001 and ∗indicates *p*-value <0.05). EV, empty vector; MOI, multiplicity of infection.

To determine the functional effects of AIP T40 phosphorylation, we stably reconstituted *Aip*^−/−^ MEFs by transducing with lentiviruses expressing AIP WT, AIP T40A, AIP T40E, and empty vector (EV) control (Fig. 4*A*). IRF7 is considered the master regulator of type I IFNs because it is IFN inducible and is responsible for a secondary and larger wave of type I IFN (37). Our previous study demonstrated that AIP suppresses type I IFN production through its IRF7 interaction and therefore *Aip*^−/−^ MEFs overproduce type I IFN and are thus highly resistant to virus infection (33). Consistently, *Aip*^−/−^ MEFs were strongly resistant to vesicular stomatitis virus (VSV)-GFP infection as measured by Incucyte S3 live-cell imaging; however, restoration of WT AIP expression restored the sensitivity of the knockout MEFs to VSV infection (Fig. 4*B*). Expression of the inactive, phosphonull AIP T40A mutant promoted resistance to VSV infection, whereas expression of the T40E phosphomimetic enhanced VSV replication (Fig. 4*B*). We next examined the expression by quantitative real-time PCR of type I IFN genes in *Aip*^−/−^ MEFs stably expressing WT AIP, T40A, or T40E. Interestingly, the basal expression of type I IFN genes (IFNα4, IFNβ) was lower in MEFs expressing WT AIP and AIP T40E compared with EV and T40A (Fig. 4*C*). In response to VSV infection, there was significantly less type I IFN in MEFs expressing WT AIP and AIP T40E (Fig. 4*D*). Together, these results indicate that T40 plays a critical role in AIP regulation of type I IFN and RNA virus replication.

### AIP T40 phosphorylation suppresses IRF7 nuclear translocation

**Figure 5:**
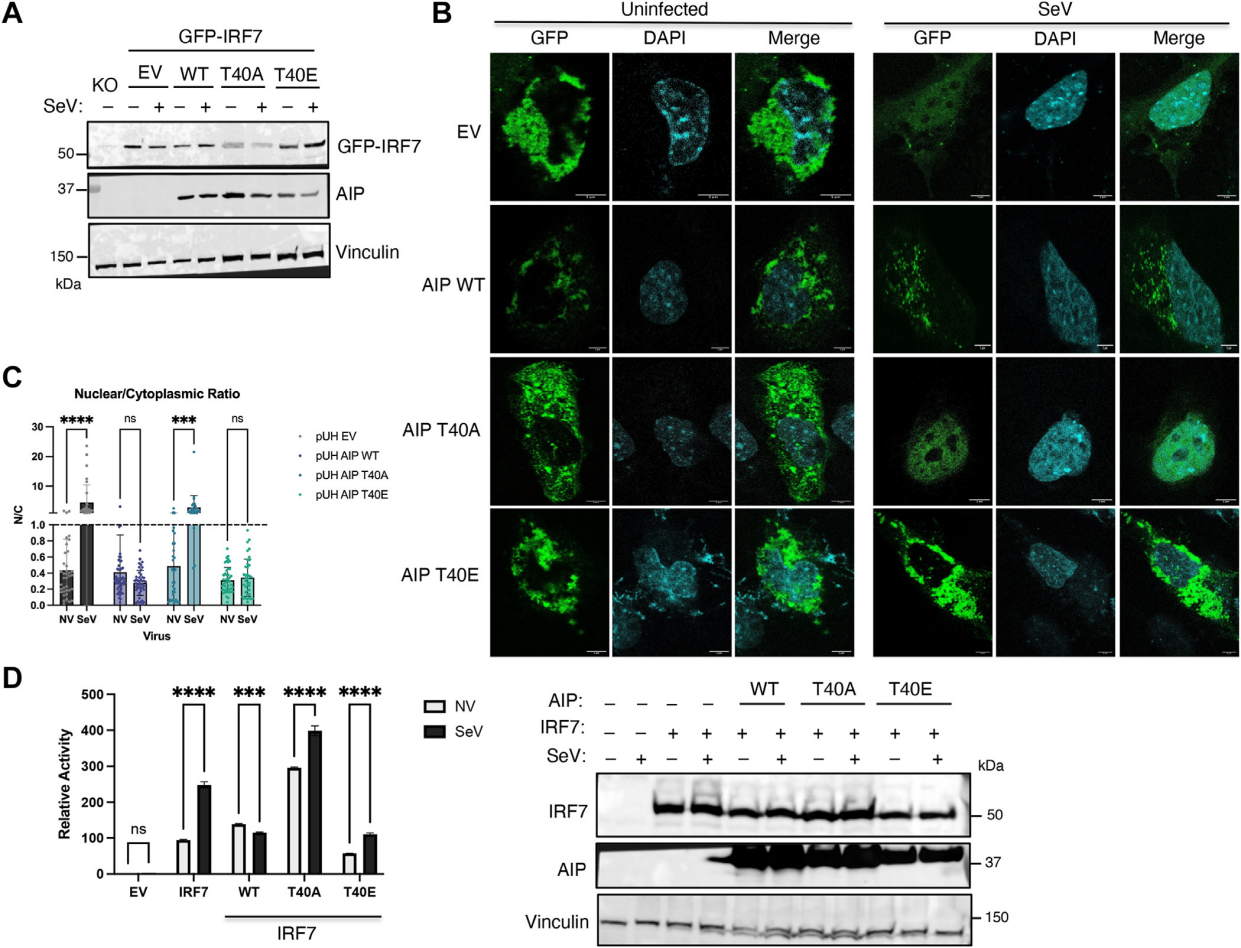
**T40 is required for AIP to inhibit IRF7 nuclear translocation.***A*–*C*, *Aip*^−/−^ MEFs stably expressing WT AIP, T40A, or T40E were transfected with GFP-IRF7. After 24 h, cells were infected with SeV for an additional 24 h. *A*, Western blot analysis of stably expressing WT AIP, T40A, or T40E transfected with GFP-IRF7. *B*, representative confocal images of stably reconstituted *Aip*^−/−^ MEFs transfected with GFP-IRF7, with or without SeV, for 24 h. Scale bars represent 5 μm. *C*, quantification of GFP-IRF7 in the nucleus, with and without SeV. N/C is the nuclear to cytoplasmic ratio. *Dots* represent individual values, and error bars represent standard deviation. ∗∗∗∗ indicates *p*-value <0.0001, ∗∗∗ indicates *p*-value <0.001, ns = not significant, n > 30. *D*, 293T cells were transfected with IFNα4-luc and pRL-TK Renilla plasmid along with the indicated Myc-AIP plasmids and/or Flag-IRF7. On the following day, cells were infected with SeV. After 24 h, cell lysates were subjected to dual luciferase assays. Protein lysates were subjected to Western blotting to confirm expression of transfected proteins.

IRF7 is normally localized in the cytoplasm, but upon virus infection IRF7 is phosphorylated, undergoes dimerization, and translocates into the nucleus where it induces the expression of type I IFN (8, 9, 10, 37, 38, 39). Our previous study demonstrated that AIP sequesters IRF7 in the cytoplasm, thus preventing type I IFN induction (33). We next sought to determine the effect of AIP T40 on virus-induced IRF7 nuclear translocation. *Aip*^−/−^ MEFs stably expressing WT AIP, T40A, or T40E were transiently transfected with GFP-IRF7 (Fig. 5*A*). We next performed confocal microscopy to examine GFP-IRF7 localization in the absence or presence of SeV infection. As expected, GFP-IRF7 was detected in the cytoplasm in MEFs lacking AIP or expressing WT AIP, T40A, or T40E (Fig. 5*B*, left; N/C < 1, Fig. 5*C*). Furthermore, in the absence of AIP, GFP-IRF7 translocated to the nucleus in SeV-infected *Aip*^−/−^ MEFs with EV (Fig. 5, *B* and *C*; N/C > 1). However, in the presence of WT AIP, GFP-IRF7 remained in the cytoplasm in SeV-infected MEFs (Fig. 5, *B* and *C*; N/C < 1). Interestingly, GFP-IRF7 translocated into the nucleus in SeV-infected MEFs expressing AIP T40A (N/C > 1) but remained in the cytoplasm in SeV-infected MEFs expressing T40E (N/C < 1) (Fig. 5, *B* and *C*).

To further assess the functional effects of AIP T40 we next performed an IFN-α4 luciferase reporter assay. Expression of Flag-IRF7 together with SeV infection led to strong activation of the IFN-α4 promoter that was blunted by expression of AIP WT (Fig. 5*D*). Similarly, overexpression of AIP T40E blocked IRF7-driven IFN-α4 reporter activation. Interestingly, AIP T40A overexpression yielded an approximately 3-fold higher basal level of IFN-α4-luciferase activity compared with IRF7 alone and IFN-α4 activity was further increased with SeV infection (Fig. 5*D*).

## Discussion

**Figure 6:**
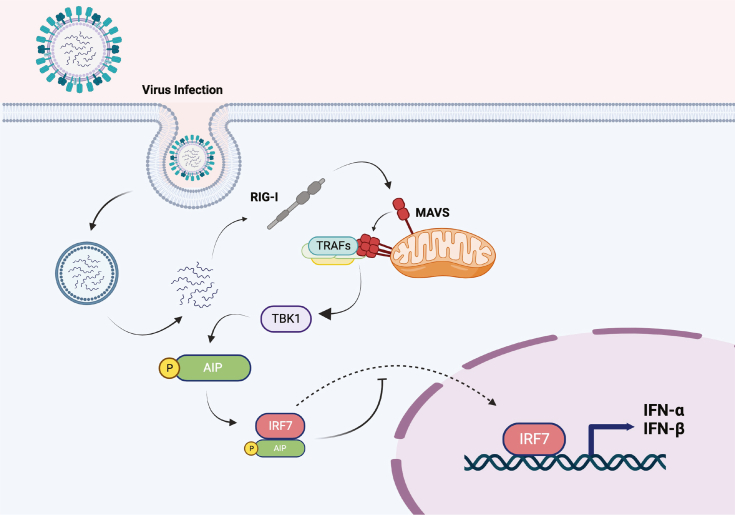
**Model depicting the proposed role of AIP in the suppression of IRF7 and innate antiviral signaling.** Following RNA virus infection, RIG-I binds to viral 5′-triphosphate dsRNA, which triggers a conformational change that exposes the caspase activation and recruitment domain (CARD) and facilitates interactions with mitochondrial antiviral signaling protein (MAVS) at the mitochondria. MAVS forms large prion-like aggregates when activated that recruit TRAF proteins, which promote activation of IKK and TBK1. Although TBK1 is a positive regulator of type I IFN by phosphorylation of IRF3, TBK1 also phosphorylates AIP at T40, which serves as a molecular switch for AIP to bind to IRF7 and prevent its nuclear translocation. AIP inhibition of IRF7 suppresses the expression of type I IFNs and interferon-stimulated genes. Image was generated using BioRender.

Our results have demonstrated a novel negative feedback mechanism that restricts IRF7 activation during virus infection. Following RNA virus infection, activated TBK1 phosphorylates AIP at T40, which promotes AIP interaction with IRF7, thus preventing its nuclear translocation (Fig. 6). As the master regulator of type I IFN, blocking IRF7 nuclear translocation and binding to the interferon-stimulated response element largely suppresses IFNα/β production. In addition to regulating AIP-IRF7 interaction, T40 also appears to regulate AIP stability, as AIP T40E increases the proteasomal degradation of AIP. AIP phosphorylation may be coupled to its destabilization as a mechanism to restore the inducibility of IRF7 activation and the inflammatory response. Immune homeostasis requires sufficient negative regulation to prevent autoimmune disease (17, 18, 19, 20, 21, 22, 23, 40, 41) balanced with immune activation to prevent severe and systemic infection (13, 14).

Upon recognition of 5′-triphosphate dsRNA, RIG-I is activated and induces a signaling cascade for type I IFN production (1, 4, 5, 6, 42). In addition to TBK1, the inducible kinase IKKi is also activated downstream of MAVS aggregation (1, 6). TBK1 and IKKi share an identical phosphorylation motif of a serine residue with leucine, isoleucine, methionine, or phenylalanine preferred at the *n* + 1 position (*n* is the site of phosphorylation); tyrosine, phenylalanine, or tryptophan at the *n* + 3 position; and tyrosine, phenylalanine, proline, or methionine at the *n* − 2 position (43, 44, 45). The AIP T40 surrounding amino acid sequences, Yx**T**LxxS, largely conform to the TBK1/IKKi consensus sequence, except for the *n* + 3 position. Our results show that SeV-induced phosphorylation of endogenous AIP is impaired in *Ikki*^−/−^ *Tbk1*^−/−^ MEFs (Fig. 1*D*), indicating critical roles of TBK1/IKKi in virus-mediated AIP phosphorylation. Although TBK1 has largely been shown to phosphorylate serine residues, it can also phosphorylate threonine residues (43). In addition to T40, S131 and S132 were also identified as TBK1-induced phosphorylation sites, and S132 phosphorylation was responsible for the band shift of AIP by TBK1 (Fig. 2*A*). Other than the leucine at *n* + 1 of S132, S131 and S132 do not conform well to the published TBK1/IKK phosphorylation motif. It is possible that S131 and/or S132 may not be direct TBK1 phosphorylation sites and may be phosphorylated by kinases functioning downstream of TBK1. Furthermore, S131 and S132 phosphomutants did not affect the AIP–IRF7 interaction or viral susceptibility (data not shown). Therefore, the phosphorylation of S131 and S132 may not play a role in regulating IRF7 and the innate immune response.

Our results indicate that AIP sequesters IRF7 in the cytoplasm and therefore inhibits IRF7 transcriptional activation of type I IFN. Another study found that the E3 ubiquitin ligase suppressor of cytokine signaling 1 (SOCS1) can interact with and inhibit IRF7; however, it showed that SOCS1 binds to IRF7 in the nucleus and triggers IRF7 degradation through its K48-linked ubiquitination (46). Therefore, there may be distinct mechanisms that promote the inhibition of cytoplasmic versus nuclear IRF7. Intriguingly, AhR has been proposed to function as a Cullin 4B E3 ligase (CUL4B) adaptor, and in a recent study AhR was shown to recruit the CUL4B–RBX E3 ligase complex to the endoplasmic reticulum innate immune adaptor protein, stimulator of interferon genes (STING), leading to the proteasomal degradation of STING in bladder cancer cells (47). In this study, a decrease in IRF7 half-life was not observed but it is possible that AIP phosphorylation could also promote IRF7 degradation since AIP T40E undergoes proteasomal degradation (Fig. 2, *D* and *F*). In support of this notion, a yeast two-hybrid screen conducted by our laboratory has revealed that AIP may interact with SOCS1 (data not shown). Future studies should address whether AIP may play a role in SOCS1-mediated ubiquitination and degradation of IRF7.

AIP was originally identified as a co-chaperone protein for AhR, acting as a brace between HSP90 and inactive AhR (27, 30). It is thought that AIP stabilizes the structure of AhR and prevents its degradation (26), with certain studies describing a 60% reduction in AhR levels in AIP-deficient cells (48). However, we did not note any difference in AhR levels or activation as measured by expression of the AhR target gene *CYP1B1* in *Aip*^−/−^ MEFs (Fig. S3, *A* and *B*). Nevertheless, AhR has been implicated as a negative regulator of innate and adaptive immunity (28) and AhR promoted the proteasomal degradation of the NF-κB transcription factor RelA/p65 in U937 macrophages treated with LPS and IFNγ (49). Further investigations are required to determine if AhR is involved in AIP-mediated suppression of IRF7 and type I IFN.

While AIP is ubiquitously expressed, its function may be tissue specific. In humans, missense mutations have been associated with 30% of familial pituitary adenomas. Many of these mutations resulted in loss of function or degradation of AIP suggesting that AIP functions as a tumor suppressor in the pituitary gland (50). However, high cytoplasmic expression of AIP was associated with a poor prognosis in pancreatic carcinoma, while nuclear AIP was associated with improved prognosis (51). In addition, high expression of AIP was associated with gastric carcinoma tumor progression and death (52). In mice, AIP was associated with increased hepatoxicity and hepatocellular damage (48). In addition, AIP is essential for cardiovascular development, such that loss of AIP is embryonically lethal (48, 53). Therefore, AIP conditional knockout mice are needed for future *in vivo* studies to determine the role of AIP in regulating IRF7 and the innate immune response to viral challenge.

Overall, we have identified TBK1-induced AIP phosphorylation at T40 as a novel negative feedback mechanism for the inhibition of IRF7. This knowledge could potentially be exploited to fine-tune the activation of IRF7 to either promote type I IFN expression in the setting of virus infections or inhibit type I IFN in certain autoimmune diseases.

## Experimental procedures

### Cells, plasmids, and reagents

293T and THP-1 cells were obtained from ATCC. *Aip*^*−/−*^ mouse embryonic fibroblasts (MEFs) were provided by Dr Auli Karhu (University of Helsinki) (54). *Ikki*^−/−^*Tbk1*^−/−^ MEFs were obtained from Dr Shizou Akira (Osaka University) (55). Myc-AIP and Flag-TBK1 plasmids were described (33, 56). Lentiviral plasmids were generated by PCR cloning AIP cDNA digested with Xba1 and BglII into the pUltraHot lentiviral plasmid (Addgene) digested with Xba1 and BamH1. Primers used for PCR are listed in Table S1. GFP-IRF7 and Flag-IRF7 were described and provided by Dr John Hiscott (Istituto Pasteur Italia) (57). IFN-α4 luciferase reporter was provided by Dr Shunbin Ning (East Tennessee State University) (58). AIP T40, S104, S131, and S132 point mutants and TBK1 K38A were generated by PCR site-directed mutagenesis (Agilent QuikChange II) using the primers listed in Table S1. CHX, Bafilomycin-A1, phorbol 12-myristate 13-acetate, MG-132, and poly(I:C) were purchased from Millipore-Sigma. Quick calf intestinal phosphatase was purchased from New England Biosciences. AIP and TBK1 recombinant proteins were purchased from Novus and Life Technologies, respectively. pUltraHot was a gift from Malcolm Moore (Addgene plasmid #24130).

### Cell culture, transfections, and luciferase assays

HEK293T cells and MEFs were cultured in Dulbecco's modified Eagle's medium supplemented with 10% heat-inactivated fetal bovine serum and 1% penicillin–streptomycin. THP-1 cells were cultured in RPMI supplemented with 10% heat-inactivated fetal bovine serum with 1% penicillin–streptomycin. THP-1 cells were differentiated by treatment with phorbol 12-myristate 13-acetate (500 ng/ml) for 48 h. DNA transfections in HEK293T cells were performed using GenJet Plus (SignaGen) according to the manufacturer’s instructions. MEF transfections were performed using Lipofectamine 3000 (Life Technologies). Lentiviruses were generated by transfecting LentiX cells with pUltraHot or pUltraHot-AIP (2500 ng), PAX (2000 ng), and VSV-G (500 ng) using GenJet (SignaGen). Two days after transfection, the supernatants were collected and virus was concentrated 100-fold using Lenti-X Concentrator (Takara) according to manufacturer’s instructions. *Aip*^−/−^ MEFs were transduced with lentivirus in the presence of 10 μg/ml polybrene (Millipore-Sigma) and sorted based on expression of mCherry. For CHX chase assays, cells were treated with 100 μg/ml CHX. For luciferase assays, cells were lysed in 1x passive lysis buffer (Promega), and luciferase activity was measured with the Dual-Luciferase assay system (Promega) according to the manufacturer’s instructions. Firefly luciferase values were normalized to *Renilla* luciferase values (internal control), and luciferase activities were presented as “relative activity” compared with nontreated control: EV or mock infection.

### Mass spectrometry

For identification of AIP phosphorylation sites with transfected AIP and TBK1, Coomassie-stained gel pieces were destained and subjected to reduction (5 mM DTT for 45 min at 60 °C) and alkylation (20 mM iodoacetamide for 20 min at room temperature in the dark). Samples were subsequently proteolyzed with 10 ng trypsin (Promega)/μl overnight at 37 °C. Dry extracted peptides after clean-up were resuspended in 8 μl 0.1% formic acid (FA). Titanium dioxide was used for phosphopeptide enrichment. Protein identification by LC-MS/MS analysis of peptides was performed using an LTQ Orbitrap Velos MS (ThermoFisher Scientific) interfaced with a nanoAcquity LC system (Waters, Corp) at the Johns Hopkins Mass Spectrometry and Proteomics Core. Peptides were fractionated by reverse-phase HPLC on a 75 μm × 15 cm PicoFrit column with a 15 μm emitter (New Objective) in-house packed with Magic C18AQ (Michrom BioResources, Inc) using 0 to 60% acetonitrile (ACN)/0.1% FA gradient over 70 min at 300 nl/min. Eluting peptides were sprayed directly into an LTQ Orbitrap Velos at 2.0 kV. Survey scans were acquired from 350 to 1800 *m/z* with up to 10 peptide masses individually isolated with a 1.9 Da window and fragmented (tandem mass spectrometry) using a collision energy of 40- and 30-s dynamic exclusion. Precursor and the fragment ions were analyzed at 30,000 and 7500 resolution, respectively. Peptide sequences were identified from isotopically resolved masses in mass spectrometry and tandem mass spectrometry spectra extracted with and without deconvolution using Thermo Scientific MS2 processor and Xtract software. Data were searched for in the human RefSeq database, with oxidation on methionine (variable), deamidation NQ (variable), phosphoSTY (variable), and carbamidomethyl on cysteine as (fixed) modifications, using Proteome Discoverer 1.3 software.

For identification of AIP phosphorylation sites with recombinant proteins, 2 μg AIP and 0.1 μg TBK1 proteins were diluted with 150 μM ATP in kinase dilution buffer VII (SignalChem) and incubated at 30 °C for 30 min. Proteins were resolved on a NuPAGE gel (ThermoFisher Scientific) and stained using colloidal blue stain (ThermoFisher Scientific) according to the manufacturer’s instructions. Bands were excised from the gel, and gel fragments were washed, dried with LCMS ACN, and incubated with 0.5 M tetraethylammonium bromide, 10 mM Tris (2-carboxyethyl) phosphine, 20 mM chloroacetamide, 2% sodium deoxycholate, pH 8.5. Proteins were reduced and alkylated with the above buffer at 80 °C for 10 min. Gel fragments were then washed and dried with ACN. Proteins in the gel were digested with 0.6 μg of Trypsin LysC (Promega) at 30 °C overnight in 0.5 M tetraethylammonium bromide. Peptides were extracted with 40% ACN in 0.1% FA and 60% ACN then purified using self-made SDB RPS tip columns (Empore made by CDS Analytical). The concentration of peptides in the eluate was quantified by measuring their absorbance at 280 nm on a Nanodrop. Samples were diluted to 0.25 mg/ml in Buffer A (water with 0.1% FA), and 1.0 μl was loaded into the mass spectrometer (TIMS Tof Flex from Bruker). The tryptic digests were injected using the NanoElute UPLC with autosampler on a Bruker Fifteen 15-cm nanoflow column with 0% ACN/water (v/v) containing 0.1% FA at a flow rate of 1 μl per minute, operating at 50 °C controlled by the Column Toaster (Bruker Daltonics), NanoElute (Bruker Daltonics). The NanoElute is coupled with TIMS quadrupole time-of-flight instrument (timsTOF Flex, Bruker Daltonics), and samples were measured in dda-PASEF phospho mode. The column emitter is housed in the nanoelectrospray source (CaptiveSpray source, Bruker Daltonics), and the source parameters were 1400 V of Capillary voltage, 3.0 l/min of dry gas, and 180 °C of dry temperature. The analytical column flow was set to 1 μl/min, and the mobile phases water/0.1% FA and ACN/0.1% FA (A and B, respectively) were applied in the linear gradients starting from 2% B and increasing to 35% in 17.22 min, followed by an increase to 95% B in 0.5 min, for 3 min, the column was equilibrated in 2% B by next 1.3 min (all % values are v/v, Water and ACN LC-MS grade solvents were purchased from Fisher Scientific). For calibration of ion mobility dimension, three ions of Agilent (622.0289, 0.9848; 922.0097, 1.1895; 1221.9906, 1.3820) spotted on a Filter were used. The sample generated Bruker.d file, was analyzed by Byonics from Protein Metrics using a UP000005640 Human database with 536 lab contaminants.

### Coimmunoprecipitation and Western blotting

Cells were lysed with RIPA buffer (50 mM Tris pH 7.4, 2% SDS, 5% glycerol) containing protease and phosphatase inhibitors (Bio-Rad), and protein concentration was estimated using the BCA protein assay kit (ThermoFisher Scientific). For coimmunoprecipitation assays, 400 μg of protein lysate was incubated overnight with mouse anti-Flag antibody (Millipore-Sigma) (1:200). On the following day, lysates were incubated with 20 μl of protein A/G PLUS-agarose beads (Santa Cruz Biotechnology) for 4 h. Beads were washed four times, and lysates were dissociated from beads according to the manufacturer’s instructions. Lysates were prepared in 4x NuPAGE LDS sample buffer (ThermoFisher Scientific) with β-mercaptoethanol reducing agent and boiled at 75 °C for 5 min. Lysates were run on 6 to 15% gradient SDS-PAGE gels and transferred onto nitrocellulose membranes using the Trans-Blot Turbo Transfer System (Bio-Rad). For Phos-Tag gels, lysates were loaded on SuperSep Phos-Tag precast gels (FujiFilm Wako Chemicals). Electrophoresis was conducted at a constant amperage of 30 mA/gel for 75 min. Before transfer, the gel was washed three times in Turbo transfer buffer with 10 mmol/L EDTA for 10 min and washed once with Turbo transfer buffer without EDTA. Antibodies are listed in Table S3. Western blots were developed with SuperSignal West Pico PLUS Chemiluminescent Substrate (ThermoFisher Scientific) and imaged with an Azure 600 Imager.

### Immunofluorescence microscopy

MEFs were cultured overnight on glass coverslips in six-well plates. After transfection and/or virus infection, cells were washed with PBS, fixed with paraformaldehyde (ThermoFisher Scientific) for 15 min, and washed three times with PBS. Cells were then permeabilized with 0.5% Triton-X in PBS for 5 min, followed by three PBS washes. Cells were blocked in 3% bovine serum albumin for 1 h. MEFs transfected with Myc-AIP plasmids were stained with mouse anti-Myc antibody (1:1000 in 3% bovine serum albumin) overnight. On the following day, cells were washed three times with PBS before incubation with Alexa Fluor 594–conjugated goat anti-mouse secondary antibody (1:1000, ThermoFisher Scientific). Coverslips were washed six times with PBS and then mounted onto slides using ProLong Diamond Antifade Mountant with DAPI staining (ThermoFisher Scientific). Images were obtained with a Leica SP8 Inverted Laser Scanning Confocal Microscope using a 63× oil objective. Images were analyzed using Fiji and JaCOP plugin for colocalization analysis. Nuclear translocation was quantified by analyzing the ratio of GFP in the nucleus to GFP fluorescence in the entire cell using Fiji.

### Quantitative real-time PCR

MEFs were seeded at 10^6^ cells/well in a six-well plate. Total RNA was isolated using the Zymo Quick RNA kit (Zymo Research). cDNA was synthesized using M-MLV reverse transcriptase (Invitrogen) according to the manufacturer’s instructions. Quantitative real-time PCR reactions were performed using 5x PowerUp SYBR Green Master Mix (Applied Biosystems); the primers are listed in Table S2. All reactions were run in triplicate on a QuantStudio 3 Real-Time PCR System (ThermoFisher Scientific) and analyzed via Design and Analysis software (Applied Biosystems). Relative gene expression was calculated by normalizing threshold cycle (ΔCt) values of genes of interest to β-Actin as described (59).

### Live cell imaging

The IncuCyte S3 live-cell analysis system (Sartorius) equipped with a 10× objective was used to measure viral replication of VSV-GFP in MEFs by enumerating cells expressing GFP relative to the total number of cells. Images were collected at 2-h intervals. Each condition was run in triplicate with 12 images collected per well. Images were collected and analyzed using IncuCyte software, and replication was normalized to green object count per phase area.

### Virus infections

Sendai virus (SeV) (Cantell strain) was purchased from Charles River Laboratories. VSV and VSV-GFP were provided by Dr Siddharth Balachandran (Fox Chase Cancer Center) (60). Cells were serum starved for 1 h, then incubated in serum-free medium containing SeV (30 HA/ml) or VSV (MOI = 1) for 1 h. Cells were replenished with complete medium following incubation with virus-containing medium.

### Sequence alignment

AIP amino acid sequences were accessed from NCBI GenBank (human, O00170; mouse, O08915; rat, Q5FWY5; bovine, Q7YRC1; chicken, Q7T048) and aligned using the ClustalW (61) algorithm within MacVector software 18.6.0.

### Statistical analysis

Statistical analysis was performed using GraphPad Prism 9.3.1. Error bars represent standard deviation of multiple samples. Two-way ANOVA with multiple comparisons was performed, and statistical significance is indicated as *p* < 0.0001 (∗∗∗∗), *p* < 0.001 (∗∗∗), *p* < 0.01 (∗∗), and *p* < 0.05 (∗).

## Data availability

All data described are contained within the document.

## Supporting information

This article contains supporting information.

## Conflict of interest

The authors declare that they have no conflicts of interest with the contents of this article.

## Figures and Tables

**Supplemental Figure S3:**