# Supplementary material for: Phosphorylation of aryl hydrocarbon receptor interacting protein by TBK1 negatively regulates IRF7 and the type I interferon response
Source: J Biol Chem. 2023 Dec 1;300(1):105525. doi: 10.1016/j.jbc.2023.105525 (PMC10792245; doi:10.1016/j.jbc.2023.105525)
Supplement: Supplemental Tables [file mmc1.pdf]

Supporting Information Table 1

Table S1. PCR Primers		
**Primer Name**	**Forward Primer**	**Reverse Primer**
AIP full length for pUltraHot (Forward: XbaI; Reverse: BglII)	5'-GATC TCTAGA ATG GCG GAT ATC ATC GCA AGA C-3'	5'-GATC AGATCT TCA ATG GGA GAA GAT CCC CCG-3'
AIP T40E Mutagenesis	5'-CAC TAC CGG GAG CTG CAC AGT GAC G-3'	3'-CGT CAC TGT GCA GCT CCC GGT AGT G-3'
AIP T40A Mutagenesis	5'- C CAC TAC CGG GCG CTG CAC AGT GAC G-3'	5'-C GTC ACT GTG CAG CGC CCG GTA GTG G-3'
AIP S131E Mutagenesis	5'- GCG TGA ACA CGA GTC CCT GGG CCA TG -3'	5'- CAT GGC CCA GGG ACT CGT GTT CAC GC -3'
AIP S131A Mutagenesis	5'- G CGT GAA CAC GCC TCC CTG GGC CAT G -3'	5'- C ATG GCC CAG GGA GGC GTG TTC ACG C -3'
AIP S132E Mutagenesis	5'- GCG TGA ACA CAG CGA GCT GGG CCA TG -3'	5'- CAT GGC CCA GCT CGC TGT GTT CAC GC -3'
AIP S132A Mutagenesis	5'- CGT GAA CAC AGC GCC CTG GGC CAT G -3'	5'- CAT GGC CCA GGG CGC TGT GTT CAC G -3'
AIP S131E/S132E Mutagenesis	5'- GCG TGA ACA CGA GGA GCT GGG CCA TG -3'	5'- CAT GGC CCA GCT CCT CGT GTT CAC GC -3'
AIP S131A/S132A Mutagenesis	5'- G CGT GAA CAC GCC GCC CTG GGC CAT G -3'	5'- C ATG GCC CAG GGC GGC GTG TTC ACG G -3'
AIP S104E Mutagenesis	5'- CTG GTG GCC AAG GAA CTC CGC AAC ATC -3'	5'- GAT GTT GCG GAG TTC CTT GGC CAC CAG -3'
AIP S104A Mutagenesis	5'- CTG GTG GCC AAG GCT CTC CGC AAC ATC -3'	5'- GAT GTT GCG GAG AGC CTT GGC CAC CAG -3
TBK1 K38A Mutagenesis	5'-GAT TTA TTT GCT ATC GCA GTA TTT AAT AAC ATA-3'	5'-TAT GTT ATT AAA TAC TGC GAT AGC AAA TAA ATC-3'

Supporting Information Table 2

Table S2. qRT-PCR Primers		
**Gene Name**	**Forward Primer**	**Reverse Primer**
Mouse β-Actin	5'- GTC CCT CAC CCT CCC AAA AG -3	5'- GCT GCC TCA ACA CCT CAA CCC -3'
Mouse IFNα4	5'- AAGCCTGTGTGATGCAGGAA -3'	5'- TGGTTGAGGAAGAGAGGGCT -3'
Mouse IFNβ	5'- ATG AAC AAC AGG TGG ATC CTC C -3	5'- AGG AGC TCC TGA CAT TTC CGA A -3'
Mouse CYP1B1	5'- CCA CCA GCC TTA GTG CAG AC -3'	5'- GGC CAG GAC GGA GAA GAG T -3'

Supporting Information Table 3

Table S3. Antibodies		
**Antibody**	**Catalog Number**	**Company**
Mouse anti-AIP	sc59730	Santa Cruz Biotechnology
Rabbit anti-AhR	sc5579	Santa Cruz Biotechnology
Mouse anti-β-actin	sc47778	Santa Cruz Biotechnology
Mouse anti-GFP	sc9996	Santa Cruz Biotechnology
Mouse anti-Flag M2	F3165	Millipore/Sigma
Mouse anti-Myc (9B11)	2276	Cell Signaling Technology
Rabbit anti-TBK1 (E8I3G)	38066	Cell Signaling Technology
Rabbit anti-IKKε/IKKi (D61F9)	3416	Cell Signaling Technology
Mouse anti-Vinculin	sc74614	Santa Cruz Biotechnology
ECL Anti-mouse IgG HRP	NA931	Cytiva
ECL Anti-rabbit IgG HRP	NA934	Cytiva
